# Supplementary material for: Identification of a Novel Variant in EARS2 Associated with a Severe Clinical Phenotype Expands the Clinical Spectrum of LTBL
Source: Genes (Basel). 2020 Sep 2;11(9):1028. doi: 10.3390/genes11091028 (PMC7563109; doi:10.3390/genes11091028)
Supplement: Supplementary file 1 [file genes-11-01028-s001.pdf]

Table S1. ***In silico*** **analysis to predict** ***EARS2*** **variants pathogenicity according to AMCG guidelines**

**Variants *EARS2* (NM_001083614.2)**	**Inheritance**	**Population frequencies**		**Conservation Scores**			**Prediction Scores**			**Functional**	
		**gnomAD**	**ExAC**	**GERP**	**PhyloP20way**	**phastCons20way**	**DANN**	**Mutation Taster**	**FATHMM**	**SIFT**	**Provean**
c.670G>A (p.Gly224Ser)	Mother	0.00087	0.00119	5.56	0.59	0.91	0.9986	DC	T	D	D
c.376C>T (p.Gln126*)	Father	-	-	5.66	0.68	0.61	0.9961	DC	D	NA	NA

DC: Disease causing; D: Damaging; T: Tolerated; NA: Not Applicable

Table S2. Clinical features in the patients reported so far harboring pathogenic variants in *EARS2*.

Author	Patients	Age of onset (months)	Disease type	Lactic acidosis	MRI brain features					Seizures	Liver involvement	Hypotony	Complexes Deficiency
					Agenesis of the corpus callosum	Cerebellar atrophy	Leukoencep halopathy	Brainstem	Thalamus				
**Steenweg, 2012** [1]	**1**	1	S	Yes	Yes	No	Yes	Yes	Yes	Yes	Yes	Yes	I, II, III and IV (Mu)<br>III and IV (Fi)
	**2**	5	M	Yes	Yes	No	Yes	Yes ✝	Yes ✝	Yes	-	Yes	-
	**3**	8	M	Yes	Yes	No	Yes	Yes ✝	Yes ✝	Yes	-	-	-
	**4**	7	M	Yes	Yes	No	Yes	Yes ✝	Yes ✝	No	-	-	-
	**5**	6	M	Yes	Yes	No	Yes	Yes ✝	Yes ✝	No	-	-	-
	**6**	4	M	Yes	Yes	No	Yes	Yes ✝	Yes ✝	No	-	-	-
	**7**	8	M	Yes	Yes	No	Yes	Yes	Yes	No	-	-	-
	**8**	10	M	Yes	Yes	No	Yes	Yes	Yes	No	-	-	-
	**9**	2.5	S	Yes	Yes	**Yes**	Yes	Yes	Yes	Yes	-	Yes	-
	**10**	<9	S	Yes	Yes	No	Yes	Yes	Yes	Yes	-	Yes	-
	**11**	1	S	Yes	Yes	No	Yes	Yes	Yes	Yes	-	Yes	-
	**12**	4	M	Yes	Yes	No	Yes	Yes	Yes ✝	No	-	-	-
**Talim, 2013** [2]	**13**	0	F	Yes	Yes	No	-	-	-	No	Yes	Yes	I and IV (Mu)
**Taylor, 2014** [3]	**14**	0	F	**-**	-	-	**No**	-	-	-	-	-	-
**Taylor, 2014** [3]	**15**	2	F	Yes	-	-	Yes	-	-	-	-	-	-
**Biancheri, 2015** [4]	**16**	3	M	Yes	Yes	-	Yes	Yes	Yes	Yes	-	Yes	I, III and IV (Mu)
**Kohda, 2016** [5]	**17**	**-**	**-**	-	-	-	-	-	-	Yes	-	-	IV (Fi)
**Kevelam, 2016** [6]	**18**	0	S	**No**	Yes ✝	No	Yes	**No**	**No**	Yes	No	Yes	-
**Danhauser, 2016** [7]	**19**	0	F	Yes	Yes	-	-	-	-	Yes	-	Yes	-
**Taskin, 2016** [8]	**20**	16	M			-	Yes	Yes	Yes	Yes	Yes	-	-
**Güngor, 2016** [9]	**21**	7	M	Yes	Yes	No	Yes	Yes	Yes ✝	Yes	No	Yes	-
**Sahin, 2016** [10]	**22**	8	S	Yes	No	-	Yes	Yes	Yes	Yes	-	-	-
**Sahin, 2016** [10]	**23**	9	**-**	-	No	-	Yes	Yes	Yes ✝	-	-	Yes	-
**Pronicka, 2016** [11]	**24**	2	**-**	Yes	-	-	-	-	-	Yes	Yes	-	I (Mu)
**Pronicka, 2016** [11]	**25**	**-**	F	Yes	-	-	-	-	-	-	-	Yes	IV (Mu)
**Oliveira, 2016** [12]	**26**	0	F	Yes	Yes	No	**No**	Yes	Yes	-	Yes	Yes	I and IV (Mu)
**Sellars, 2017** [13]	**27**	3	S	Yes	-	-	-	-	Yes	-	Yes	Yes	-
**Balushi, 2019** [14]	**28**	2	-	Yes	No	-	Yes	-	-	No	Yes	-	-
**Prasun, 2019** [15]	**29**	3	S	Yes	No	-	Yes	**No**	**No**	Yes	Yes	No	-
**Our patient, 2020**	**30**	12	S	Yes	Yes	**Yes**	Yes	-	-	Yes	No	No	I and III (Mu)<br>III (Fi)

M – Moderate; S – Severe; F – Fatal; MRI: Magnetic Resonance Imaging; Mu – muscle; Fi – fibroblasts; ✝ *- condition improvement*

Table S3 - Genes included in the Mitochondrial gene panel

AARS2 ABCB11 ACAD8 ACAD9 ADCK3 AFG3L2 AGK AIFM1 APOPT1 APTX ATAD3A ATP5A1 ATP5E ATP5F1D ATPAF2 BCS1L BOLA3 C10ORF2 C12ORF65 CARS2 COA5 COA6 COQ2 COQ4 COQ5 COQ6 COQ7 COQ9 COX10 COX11 COX14 COX15 COX6B1 COX8A CPS1 CYC1 CYCS DARS2 DGUOK DLAT DNA2 DNM1L E4F1 EARS2 ELAC2 FAM36A FARS2 FARSB FASTKD2 FBXL4 FOXRED1 GARS GFM1 GFM2 GTPBP3 GYG2 HARS2 HCCS HIBCH HSPD1 HSPE1 ISCA1 ISCA2 LARS2 LONP1 LRPPRC LYRM4 LYRM7 MARS2 MDH2 MFF MFN1 MFN2 MGME1 MIPEP MPV17 MRPL12 MRPL3 MRPL44 MRPS16 MRPS22 MRPS23 MRPS34 MTFMT MTO1 MTPAP NARS2 NAXE NDUFA1 NDUFA10 NDUFA11 NDUFA12 NDUFA13 NDUFA2 NDUFA3 NDUFA4 NDUFA4L2 NDUFA5 NDUFA9 NDUFAF1 NDUFAF2 NDUFAF3 NDUFAF4 NDUFAF5 NDUFAF6 NDUFB1 NDUFB10 NDUFB11 NDUFB2 NDUFB3 NDUFB4 NDUFB5 NDUFB6 NDUFB7 NDUFB8 NDUFB9 NDUFS1 NDUFS2 NDUFS3 NDUFS4 NDUFS5 NDUFS6 NDUFS7 NDUFS8 NDUFV1 NDUFV2 NDUFV3 NFS1 NFU1 NUBPL OPA1 PARS2 PC PDHA1 PDHA2 PDHB PDHX PDP1 PDSS1 PDSS2 PET100 PET117 PGAP2 PMPCB PNPLA4 PNPT1 POLG POLG2 QRSL1 RMND1 RNASEH1 RRM2B SARS SARS2 SCO1 SCO2 SDHA SDHAF1 SDHD SFXN4 SLC25A12 SLC25A26 SLC25A4 SLC25A42 SLC25A46 SUCLA2 SUCLG1 SURF1 TACO1 TARS2 TAZ TBCD TBCE TFAM TK2 TMEM126B TMEM70 TRAK1 TRIT1 TRMT10C TRMT5 TUFM TXN2 TYMP UQCC3 UQCRB UQCRC2 UQCRQ VARS2 VDAC1 VDAC2 WARS2

References

1. Steenweg, M.E.; Ghezzi, D.; Haack, T.; Abbink, T.E.M.; Martinelli, D.; van Berkel, C.G.M.; Bley, A.; Diogo, L.; Grillo, E.; Te Water Naudé, J.; et al. Leukoencephalopathy with thalamus and brainstem involvement and high lactate "LTBL" caused by EARS2 mutations. *Brain* **2012**, *135*, 1387–1394, doi:10.1093/brain/aws070.

2. Talim, B.; Pyle, A.; Griffin, H.; Topaloglu, H.; Tokatli, A.; Keogh, M.J.; Santibanez-Koref, M.; Chinnery, P.F.; Horvath, R. Multisystem fatal infantile disease caused by a novel homozygous EARS2 mutation. *Brain* **2013**, *136*, e228, doi:10.1093/brain/aws197.

3. Taylor, R.W.; Pyle, A.; Griffin, H.; Blakely, E.L.; Duff, J.; He, L.; Smertenko, T.; Alston, C.L.; Neeve, V.C.; Best, A.; et al. Use of whole-exome sequencing to determine the genetic basis of multiple mitochondrial respiratory chain complex deficiencies. *JAMA* **2014**, *312*, 68–77, doi:10.1001/jama.2014.7184.

4. Biancheri, R.; Lamantea, E.; Severino, M.; Diodato, D.; Pedemonte, M.; Cassandrini, D.; Ploederl, A.; Trucco, F.; Fiorillo, C.; Minetti, C.; et al. Expanding the Clinical and Magnetic Resonance Spectrum of Leukoencephalopathy with Thalamus and Brainstem Involvement and High Lactate (LTBL) in a Patient Harboring a Novel EARS2 Mutation. *JIMD Rep* **2015**, *23*, 85–89, doi:10.1007/8904_2015_434.

5. Kohda, M.; Tokuzawa, Y.; Kishita, Y.; Nyuzuki, H.; Moriyama, Y.; Mizuno, Y.; Hirata, T.; Yatsuka, Y.; Yamashita-Sugahara, Y.; Nakachi, Y.; et al. A Comprehensive Genomic Analysis Reveals the Genetic Landscape of Mitochondrial Respiratory Chain Complex Deficiencies. *PLOS Genetics* **2016**, *12*, e1005679, doi:10.1371/journal.pgen.1005679.

6. Kevelam, S.H.; Klouwer, F.C.C.; Fock, J.M.; Salomons, G.S.; Bugiani, M.; van der Knaap, M.S. Absent Thalami Caused by a Homozygous EARS2 Mutation: Expanding Disease Spectrum of LTBL. *Neuropediatrics* **2016**, *47*, 64–67, doi:10.1055/s-0035-1568987.

7. Danhauser, K.; Haack, T.B.; Alhaddad, B.; Melcher, M.; Seibt, A.; Strom, T.M.; Meitinger, T.; Klee, D.; Mayatepek, E.; Prokisch, H.; et al. EARS2 mutations cause fatal neonatal lactic acidosis, recurrent hypoglycemia and agenesis of corpus callosum. *Metab Brain Dis* **2016**, *31*, 717–721, doi:10.1007/s11011-016-9793-2.

8. Taskin, B.D.; Karalok, Z.S.; Gurkas, E.; Aydin, K.; Aydogmus, U.; Ceylaner, S.; Karaer, K.; Yilmaz, C.; Pearl, P.L. Early-Onset Mild Type Leukoencephalopathy Caused by a Homozygous EARS2 Mutation. *J Child Neurol* **2016**, *31*, 938–941, doi:10.1177/0883073816630087.

9. Güngör, O.; Özkaya, A.K.; Şahin, Y.; Güngör, G.; Dilber, C.; Aydın, K. A compound heterozygous EARS2 mutation associated with mild leukoencephalopathy with thalamus and brainstem involvement and high lactate (LTBL). *Brain Dev.* **2016**, *38*, 857–861, doi:10.1016/j.braindev.2016.04.002.

10. Şahin, S.; Cansu, A.; Kalay, E.; Dinçer, T.; Kul, S.; Çakır, İ.M.; Kamaşak, T.; Budak, G.Y. Leukoencephalopathy with thalamus and brainstem involvement and high lactate caused by novel mutations in the EARS2 gene in two siblings. *J. Neurol. Sci.* **2016**, *365*, 54–58, doi:10.1016/j.jns.2016.04.008.

11. Pronicka, E.; Piekutowska-Abramczuk, D.; Ciara, E.; Trubicka, J.; Rokicki, D.; Karkucińska-Więckowska, A.; Pajdowska, M.; Jurkiewicz, E.; Halat, P.; Kosińska, J.; et al. New perspective in diagnostics of mitochondrial disorders: two years' experience with whole-exome sequencing at a national paediatric centre. *J Transl Med* **2016**, *14*, doi:10.1186/s12967-016-0930-9.

12. Oliveira, R.; Sommerville, E.W.; Thompson, K.; Nunes, J.; Pyle, A.; Grazina, M.; Chinnery, P.F.; Diogo, L.; Garcia, P.; Taylor, R.W. Lethal Neonatal LTBL Associated with Biallelic EARS2 Variants: Case

Report and Review of the Reported Neuroradiological Features. *JIMD Rep* **2016**, *33*, 61–68, doi:10.1007/8904_2016_581.

13. Sellars, E.A.; Balmakund, T.; Bosanko, K.; Nichols, B.L.; Kahler, S.G.; Zarate, Y.A. Severe Metabolic Acidosis and Hepatopathy due to Leukoencephalopathy with Thalamus and Brainstem Involvement and High Lactate. *Neuropediatrics* **2017**, *48*, 108–110, doi:10.1055/s-0036-1593984.

14. Balushi, A.A.; Matviychuk, D.; Jobling, R.; Salomons, G.S.; Blaser, S.; Mercimek-Andrews, S. Phenotypes and genotypes of mitochondrial aminoacyl-tRNA synthetase deficiencies from a single neurometabolic clinic. *JIMD Reports* **2020**, *51*, 3–10, doi:10.1002/jmd2.12079.

15. Prasun, P.; Mintz, C.; Cork, E.; Naidich, T.P.; Webb, B.D. Broad spectrum of clinical presentation in EARS2 beyond typical "leukoencephalopathy with thalamus and brain stem involvement." *J. Neurol. Sci.* **2019**, *406*, 116448, doi:10.1016/j.jns.2019.116448.
